# The interplay between cognition, functional and dual-task gait in persons with a vestibular disorder versus healthy controls

**DOI:** 10.1038/s41598-023-35904-z

**Published:** 2023-06-22

**Authors:** Marousa Pavlou, Sergi G. Costafreda, William Galsworthy, George Korres, Doris-Eva Bamiou

**Affiliations:** 1 Centre for Human and Applied Physiological Sciences, School of Basic and Medical Biosciences, Faculty of Life Sciences and Medicine, King’s College London, Shepherd’s House, Guy’s Campus, London, SE1 1UL UK; 2 Department of Neuro-Otology, University College London Hospitals, London, UK; 3 Ear Institute, University College London, London, UK; 4 Biomedical Research Centre, University College London Hospitals, London, UK

**Keywords:** Neuroscience, Physiology, Medical research, Neurology, Risk factors

## Abstract

Close links exist between vestibular function and cognition. Dual-task (DT) tests may have ecological validity to assess the impact of daily life cognitive-motor demands in people with vestibular dysfunction (PwVD), functional gait and falls risk. The present paper aimed at building predictive models for functional gait under DT conditions, while clarifying the impact of vestibular dysfunction, individual characteristics, varying task types and motor-cognitive demands. Case-controlled observational study with 39 PwVD and 62 healthy participants. The Functional Gait Assessment (FGA), with and without an additional motor, numeracy, or literacy task, was completed. Multiple linear regression was used to fit models to predict FGA under single and DT performance. Dual task cost (DTC, %) was calculated to assess DT interference on FGA performance using the equation: 100*(single task score–dual task score)/single-task score. Following Bonferroni corrections for multiple comparisons (corrected alpha level of 0.003), PwVD had poorer performance than controls for all FGA conditions (*p* < 0.001), motor (− 3.94%; *p* = 0.002) and numeracy (− 22.77%; *p* = 0.001) DTCs and spatial working memory (*p* = 0.002). The literacy DTC was marginally significant (− 19.39% *p* = 0.005). FGA single and DT motor, numeracy, and literacy models explained 76%, 76%, 66% and 67% of the variance respectively for PwVD. Sustained attention, visual memory and sex contributed to all models; short-term visual recognition memory, balance confidence, and migraine contributed to some models. Cognitive performance is impaired in PwVD. Motor, numeracy and literacy tasks impair functional gait performance. Cognitive assessment and FGA with a numeracy or literacy cognitive component should be included within assessment protocols and considered in the provision of targeted interventions for PwVD.

## Introduction

People with vestibular dysfunction (PwVD) often report cognitive symptoms including memory loss, poor concentration, “brain fog” and an inability to multitask^1^.

Human and animal studies have demonstrated that vestibular disorders are associated with cognitive dysfunction, most prominently in visuospatial and attentional domains but also in executive function, memory, and processing speed^1–3^.

Cognitive rather than mobility tasks more strongly predict activities of daily living impairments in PwVD, and cognitive impairments have a direct impact on activity limitation^4^. Intriguingly, there is extensive reciprocal connectivity between vestibular and cortical and subcortical areas, raising the possibility of causal links^5^. However, the exact mechanism that underpins vestibular and cognitive associations remains unclear. Potential mechanisms, not necessarily mutually exclusive, include cortical network atrophy driven by vestibular loss, and involving the hippocampus^6–8^; a top down higher order regulation of vestibular functions by the parieto-temporal and parietal cortex^9^ that is necessary for spatial representation; the well-established attentional requirements of both static and dynamic balance^10^; and the presence of bidirectional interactions between psychological state, affective disorders and vestibular symptoms^11^.

These close interactions are illustrated in the cognitive-motor interference (CMI)^12,13^ phenomenon, characterized by the deterioration in performance of a cognitive and/or motor task when both are performed simultaneously. CMI can be assessed using a dual-task (DT) testing paradigm^7^. It is well-established that PwVD require additional attentional resources to maintain balance, thus probably limiting cognitive resources available for other tasks^1^.

DT studies in PwVD that use “static” sitting or standing balance tasks report varied outcomes ranging from a greater decline in cognitive task performance^9^ for PwVD versus healthy controls to no difference between groups for either task^10^; in contrast, the few studies which include a dynamic motor task (i.e. gait) consistently show greater decline in either the motor or cognitive DT for PwVD versus healthy participants^8^. Dynamic tasks may thus be more sensitive in prompting CMI in PwVD and the complexity of both tasks is crucial^14^.

Currently CMI in PwVD has only been assessed with DT paradigms incorporating a single, cognitive and motor task. This setting does not represent real-life situations typically involving diverse complex tasks performed during multifaceted gait e.g., talking while avoiding obstacles. Furthermore, no studies in PwVD have considered the effect of confounders such as subjective symptom intensity, cognitive function, and hearing loss on DT performance. In PwVD, lower balance confidence, worse self-perception of handicap and low mood are associated with poorer performance on functional gait measures, deterioration in measurable gait parameters, and/or higher fall incidence^15–18^. In healthy adults, worse hearing capacity is associated with poorer performance on the Functional Gait Assessment^19^, but no studies to date have assessed this in PwVD.

Primary aims of the current study were to extend the DT paradigm to include a standardized complex gait assessment (Functional Gait Assessment, FGA^20^) in combination with a motor, numeracy and literacy task to a) investigate the effect of various task types and categories on FGA performance and falls risk and b) develop predictive models for single and DT FGA performance in PwVD versus healthy controls. Secondary study aims were to compare cognitive performance between PwVD versus healthy controls on an extended set of visual processing tests, and identify associations between cognitive domains, dynamic gait with and without DTs, and subjective symptoms, psychological state, and demographic variables.

## Methodology

### Study design, standard protocol approvals, and patient consent

A regional ethical standards committee on human experimentation approved this case-controlled, observational study (Reference: 09/H0718/29) with a priori analysis involving collecting data directly from participants. Strengthening the Reporting of Observational Studies in Epidemiology (STROBE) reporting guidelines were followed^21^. Written informed consent was obtained from all participants.

### Participants

Between 2016 and 2019, PwVD were recruited from neuro-otology clinics at the National Hospital for Neurology and Neurosurgery, Queen Square UK, after a complete neurological and neuro-otological examination, including Hallpike positional testing, pure-tone air-conduction and bone conduction threshold audiometry with and without masking, electronystagmography, and caloric testing. In persons with recurrent headaches, migraine was diagnosed according to the International Headache Society International Classification of Headache Disorders 3rd edition diagnostic criteria^22^; vestibular migraine (VM) was diagnosed if symptoms fit Bárány society criteria^23^. Inclusion criteria were (1) clinical diagnosis of a peripheral vestibular disorder, (2) chronic dizziness and/or unsteadiness, (3) age 18 to 80 years old, and (4) no completion of a vestibular rehabilitation program. Patients with (1) central nervous system involvement, excluding migraine diagnosed as per the International Headache Society International Classification of Headache Disorders 3rd edition diagnostic criteria^22^; (2) fluctuating symptoms, for example, active Ménière’s disease; and (3) blindness; (4) severe or profound hearing loss in the better hearing ear (i.e., average of pure tone hearing threshold levels at 250, 500, 1000, 2000 and 4000 Hz that exceeds 71 decibels hearing level) and (5) orthopedic deficit affecting balance and gait were excluded. Patients with severe migraine (> 3 migraine headaches monthly) or severe depression (Hospital Anxiety and Depression (HADS) score ≥ 15/21^24^) were also excluded.

Independently mobile, healthy participants, aged 18–80 years old, were recruited via posters placed in local community centers and circular university email to staff and students. Exclusion criteria, chosen due to their potential impact on FGA and/or cognitive test performance, included previous diagnosis of a neurological or vestibular disorder, hearing loss, migraine, cognitive impairment and/or orthopedic/musculoskeletal disorder affecting balance and/or gait.

### Outcome measures

The primary outcome to be predicted was the FGA^20^ total score which assesses performance on complex gait tasks (i.e. walking with changes in speed, head turns or stepping over obstacles). The FGA total score ranges from 0 to 30 with higher scores indicating better performance. It has shown to be reliable and valid for use in PwVD^20^. Secondary outcomes to be predicted were the FGA DT conditions. The FGA assessment was completed four times in total for each participant: in isolation (FGA single) and while simultaneously performing a motor (FGA-M), literacy (FGA-L) or numeracy (FGA-N) task. The original FGA was always completed first, followed by the DT FGA in computer generated random order (www.randomizer.org). The FGA-M was performed using the dominant hand to hold a half full cup of water with the elbow flexed at 90°. The cognitive FGA-N DT involved a. counting backwards from 100 in 7’s, b. reciting the 8-multiplication table and c. reciting 7 division tables. The cognitive FGA-L DT involved reciting alternate a. alphabet letters, b. days in a week and c. months in a year. Participants performed the cognitive DTs in the order of a → b → c → a. When repeating the same task, participants started from the number, alphabet, day, or month where they finished the previous time.

### Predictors

Predictor variables were identified by the clinical experts based on expert clinical knowledge^25^ and choices were supported by scientific evidence which demonstrates an association between the predictor variable with gait performance and/or DT ability. These variables included age, sex, diagnosis, hearing loss, migraine history, cognitive function measures, subjective symptoms, and psychological state^15,16,18,19,26–29^. Each participant completed the following tests and self-report measures.

The *Cambridge Neuropsychological Test Automated Battery (CANTAB)* software (Cambridge Cognition, Cambridge, UK)^30^, a semiautomated computer program that employs touch screen technology, assessed cognitive function. The cognitive tests performed were rapid visual information processing (RVP); paired associates learning (PAL); spatial working memory (SWM), reaction time (RTI), and delayed matching to sample (DMS). A brief description of each test, what it measures, and data obtained is included in Table 1.

**Table 1:** Brief description of CANTAB cognitive function tests.

	RVP	PAL	SWM	RTI	DMS
Measures	Sustained attention; Dysfunction in parietal and frontal lobe; Sensitive to general cognitive performance	Visual memory and new learning; Changes in medial temporal lobe functioning; Sensitive to mild cognitive impairment, age related memory loss	Retain spatial information; Manipulation remembered items in working memory; Sensitive to frontal lobe and executive dysfunction	Motor and mental response speeds to visual target	Choice recognition memory for abstract patterns; Sensitive to damage in medial temporal area; Assess short term visual memory and simultaneous matching
Time	10 min	10 min	8 min	5 min	10 min
Task	Numbers from 2 to 9 appear in a random order (rate 100 digits per minute) Detection of target sequence (i.e. 2-4-6, 3-5-7, 4-6-8) via touchscreen	Boxes are displayed and ‘opened’ in randomized order. One or more of the boxes will contain a pattern Different patterns are displayed in the middle of the screen and the original location of patterns in box to be detected	Boxes displayed on screen By process of elimination finding of one single blue token per box followed by manipulative task	Touching a given circle on screen as soon as yellow dot appears Modes: predictable (single circle) Unpredictable (five circles)	Display of abstract pattern on screen, after delay (0, 4 or 12 s) representation of different patterns Recognition of first demonstrated pattern
Outcome measures Included in analysis	Median latency (speed of response in ms; higher scores indicate poorer performance)	Total errors adjusted (higher scores indicate poorer performance)	Strategy (the number of unique boxes from which a participant starts a new search in the 6 and 8 box trials; higher scores indicate poorer performance)	Median simple reaction time (amount of time in ms to release the response button after the presentation of the target stimulus; longer duration indicates poorer performance)	Percent correct (lower % indicates poorer performance)

RVP, rapid visual processing; PAL, paired associate learning; SWM, spatial working memory; RTI, reaction time; DMS, delayed matching to sample; ms, milliseconds.

All participants completed validated self-report measures regarding symptoms, symptom triggers, balance confidence and psychological state. The Activities-specific Balance Confidence scale (ABC) assesses balance confidence in 16 daily activities with various difficulty levels^31^. Scores range from 0 (no confidence) to 100 (complete confidence). The HADS is a 14-item scale consisting of two subscales to assess symptoms of anxiety disorders (HADS-A) and depression (HADS-D)^32^. Scores between 8-10/21 indicate mild, 10-14/21 moderate and 15-21/21 severe depression or anxiety symptoms, respectively^24^. The Vertigo Symptom Scale (VSS) assesses frequency and severity of common vestibular (VSS-V; e.g. vertigo, imbalance) and autonomic/somatic (VSS-A; e.g. heart pounding, heavy feeling in the arms or legs) symptoms^33^. The Situational Characteristics Questionnaire (SCQ) measures symptom provocation or exacerbation frequency in environments with visual-vestibular conflict or intense visual motion (i.e., crowds)^34^. The Dizziness Handicap Inventory (DHI) is a 25-item scale which quantifies the impact of dizziness on ADLs by evaluating self-perceived handicap in PwVD and includes an emotional (DHI-E), functional (DHI-F) and physical (DHI-P) domain^35^. Total score ranges from 0 to 100 with higher score indicating greater handicap^35^. Only the total DHI score was considered as a predictor variable as it is more reliable than scores for individual subscales^36,37^.

### Statistical analysis

IBM SPSS version 26 (IBM Corp, Armonk, New York, USA) was used for statistical analysis. All data are presented as mean ± SD and median and interquartile range. The data was analyzed using a variety of statistical tests and techniques. First, the chi-square test and Mann–Whitney U test were performed to examine how demographic information differed between the study groups. To test the normality of the distribution for variables, the Shapiro–Wilk test, histogram, and Q-Q plots were used. The results indicated that the data was not normally distributed, therefore, the Mann–Whitney non-parametric test was selected to compare variables between study groups. The effect size was calculated for the Mann–Whitney U test, the standardized test statistic z is divided by the square root of the number of pairs (n). In addition, multiple comparisons can increase the likelihood of Type I errors^38^, and therefore, it is important to adjust p-values to control for such errors. In this study, 19 Mann–Whitney U tests were conducted on a single group variable. To account for multiple comparisons, a Bonferroni correction was applied. The corrected alpha level was calculated by dividing the desired overall alpha level (0.05) by the number of tests performed (19), resulting in a new corrected alpha level of 0.003.

Within-group DTC differences were assessed using Wilcoxon signed-rank test. Spearman’s correlation assessed for relationships between cognitive performance, self-report measures and demographic variables (age and sex for both groups and migraine history and hearing loss only for PwVD).

The dual task cost (DTC) was calculated to assess DT interference. DTC is the percentage change in FGA performance due to the DT condition and was calculated separately for FGA-M, FGA-N and FGA-L using the following equation^39^:

$$ {\text{DTC}}\,(\% ) = 100 \, *\frac{{\left( {{\text{DT}}{-}{\text{Single}}\,{\text{task}}} \right)}}{{{\text{Single}}\,{\text{task}}}} $$

In the current study, a more negative DTC indicates a higher impact of adding a secondary task on the primary task (FGA), as a higher FGA score is better. The DTC was only calculated for the primary motor task (FGA), but not for secondary numeracy and literacy task performance as baseline performance on these tasks was not collected.

Predictive models were developed under a multiple linear regression modelling framework as outcomes were continuous. A backwards selection approach was applied to a full model including all potentially relevant predictors that met assumption criteria to derive FGA single and DT performance models. Assumption criteria were (a) independence of residuals (Durbin Watson test values 1.5–2.5/4); (b) linearity, assessed by partial regression plots and a studentized residuals against predicted values plot; (c) homoscedasticity, assessed by visual inspection of a studentized residuals versus unstandardized predicted values plot; (d) no multicollinearity assessed by tolerance values > 0.1 and no correlations between predictors > 0.7; (e) no significant outliers, assessed by checking for studentized deleted residuals >  ± 3 SD, leverage values > 0.2, and values for Cook's distance > 1; and (f) assumption of normality was met, assessed by a Q-Q Plot^40^. If highly correlated predictors were identified, only one was included in the multivariable modelling; outliers were filtered out of the data set and the multiple regression analysis was re-run. Model performance was evaluated by calculating adjusted R^2^. Significance level was set at 0.05.

### Ethics approval

Approval was obtained from the ethics committee of the London-Central National Health Service Research Authority (Reference: 09/H0718/29). The procedures used in this study adhere to the tenets of the Declaration of Helsinki.

### Consent to participate

Informed consent was obtained from all individual participants included in the study.

## Results

### Demographics

**Figure 1:**
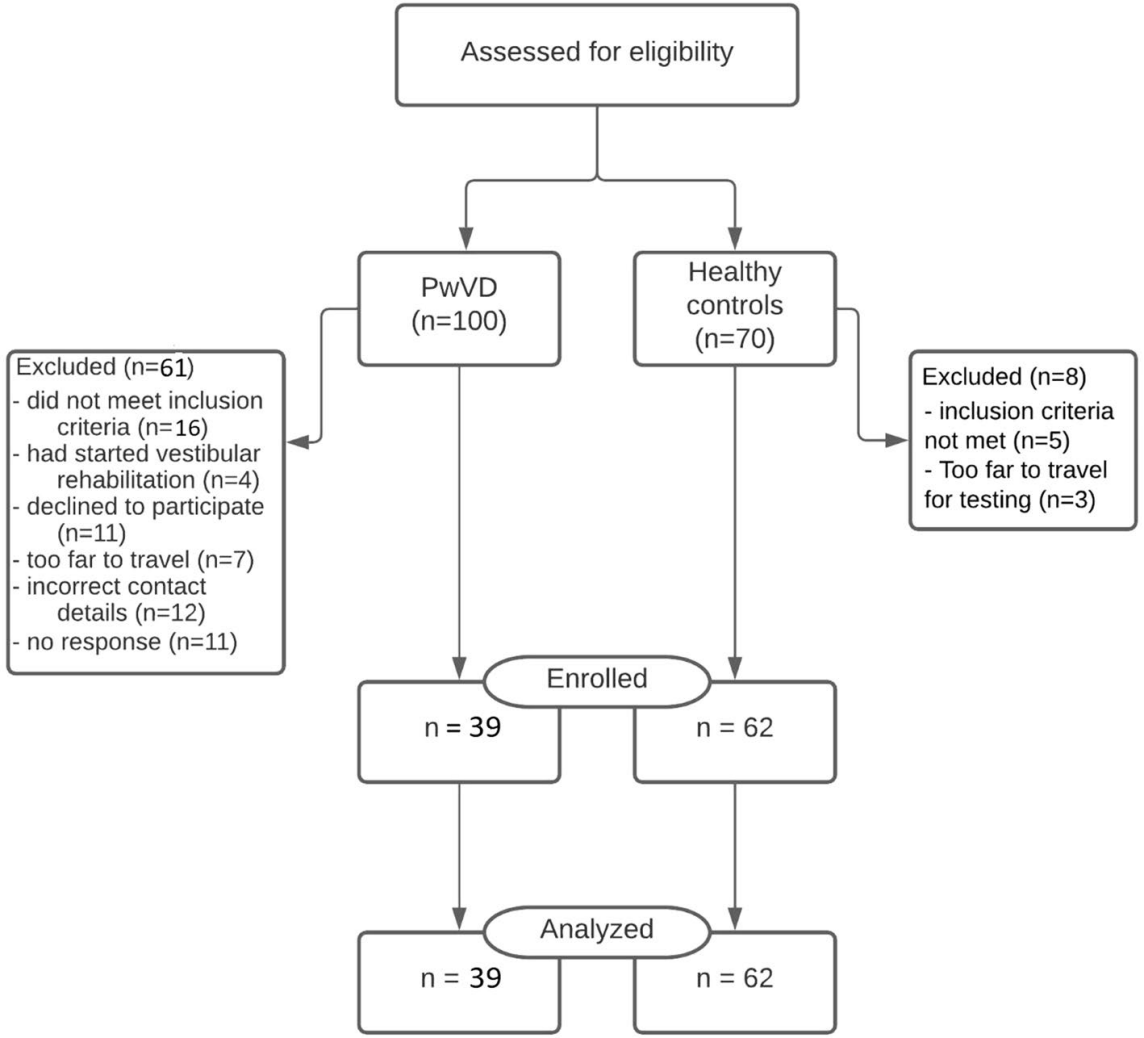
Flow diagram of the study regarding numbers examined for eligibility, confirmed eligible, included in the study, and analyzed.

Thirty-nine PwVD and 62 healthy participants were recruited. Figure 1 summarizes the flow of participants through the study. In PwVD, 48.72% had a migraine history and 33.3% had hearing loss. Demographic data, including vestibular diagnoses, are in Table 2.

**Table 2:** Participant characteristics.

Variable	Healthy participants	People with a vestibular disorder
Age (y) (mean, range)	51.77 (23–74)	50.69 (25–74)
Sex (n)	62	39
Female (n, %)	35 (56.45%)	27 (67.50%)
Male (n, %)	27 (43.55%)	14 (35.89%)
Presence of migraine (n, %)	0	19 (48.72%)
Hearing loss (n, %)	0	13 (33.3%)
		Unilateral (5)
		Bilateral (8)
Diagnosis (n)		VN (18)
		VM (12)
		VN (+M) (7)
		Acoustic neuroma (2)

VN, vestibular neuritis; VM, vestibular migraine; M, migraine.

### FGA single and DT

Between-group differences were observed for mean FGA single (*U* = 158.00, *z* = − 7.58, *p* < 0.001, *r* = 0.74), motor (*U* = 122.50, *z* = − 7.66, *p* < 0.001, *r* = 0.76), literacy (*U* = 345.50,* z* = − 6.04, *p* < 0.001, *r* = 0.60) and numeracy (*U* = 361.00. *z* = − 5.93, *p* < 0.001, *r* = 0.54) scores (Table 3), with poorer performance in PwVD. DTCs were also increased in PwVD relative to controls for FGA motor (*U* = 767.50. *z* = − 3.17, *p* = 0.002, *r* = 0.32), literacy (*U* = 807.50, *z* = − 2.80, *p* = 0.005, *r* = 0.28) and numeracy (*U* = 743.50, *z* = − 3.25, *p* = 0.001, *r* = 0.32) (Table 3)**.** When the Bonferroni correction is applied, the between-group difference for DTC is significant only for FGA motor and FGA numeracy.

**Table 3:** Comparison of performance between healthy participants and people with a vestibular disorder on various variables.

	Healthy participants (*n* = 62)		People with a vestibular disorder (*n* = 39)		*P*
	Mean (SD)	Mdn (range)	Mean (SD)	Mdn (range)	
FGA	28.81 (1.72)	30 (28,30)	21.62 (5.56)	24 (19,25)	< 0.001
FGA-M	28.61 (1.67)	29 (28,30)	20.80 (5.8)	23 (17,25)	< 0.001
FGA-N	24.95 (5.18)	27 (23,28)	17.1 (6.5)	18 (13,22)	< 0.001
FGA-L	25.47 (3.9)	26 (24,28)	17.83 (6.44)	19 (15,23)	< 0.001
FGAM-DTC	− 0.2 (4.87)	0 (− 3.33,1.67)	− 3.94 (7.61)	− 4 (− 10.28,0)	0.002
FGA-N-DTC	− 13.75 (15.99)	− 10 (− 20,− 3.33)	− 22.77 (19.75)	− 20 (− 30.68,− 10.28)	0.001
FGA-L-DTC	− 11.18 (12.69)	− 7.02 (− 16.67,− 3.33)	− 19.39 (19.03)	− 15.89 (− 28,− 6.79)	0.005
SCQ	0.18 (0.21)	0.13 (0,0.26)	1.81 (1.11)	2 (0.58,2.68)	< 0.001
VSS-V	0.05 (0.11)	0 (0,0.05)	1.18 (0.9)	0.89 (0.53,1.63)	< 0.001
VSS-A	0.33 (0.32)	0.3 (0.07,0.53)	1.45 (0.87)	1.47 (0.67,2.13)	< 0.001
DHI-Total	0.9 (2.84)	0 (0,0)	55.54 (23.66)	60 (34,74)	< 0.001
ABC	95.1 (6.3)	97.5 (93,99)	60.58 (28.39)	63.13 (34,86)	< 0.001
HADS-A	3.82 (3.28)	3 (1,6)	9.21 (5.66)	9 (4,14)	< 0.001
HADS-D	1.71 (1.77)	1 (0,3)	6.97 (4.84)	6 (3,10)	< 0.001
DM	86.93 (9.22)	87.5 (83.33,93)	83.68 (11.52)	83.33 (75,91.67)	0.147
PAL	17.02 (15.86)	12 (5,23)	23.74 (18.6)	18 (10,34)	0.029
RTI	297.41 (53.49)	289.75 (251,341)	324.51 (66.58)	310 (288,345)	0.036
SWM	22.45 (9.85)	23.5 (15,31)	28.26 (6.43)	30 (22,33)	0.003
RVP	435.6 (94.54)	427 (360.5,485.5)	461.92 (117.57)	449.5 (386,504)	0.246

Values are presented as M (SD) and Mdn (Range). Mann–Whitney U tests were used to compare variables between groups. The "Range" column presents the interquartile range (*IQR*), which represents the 25th percentile (*Q*1) and the 75th percentile (*Q*3) of the distribution. The SD and Range are presented in parentheses. *M*, Mean; *SD*, Standard Deviation; *Mdn*, Median. Corrected alpha level *p* = 0.003.

Substantial within-group differences were observed for FGA single with FGA-L (Healthy: *Z* = − 5.99, *p* < 0.001; PwVD: Z = − 5.12, *p* < 0.001) and FGA-N (Healthy: *Z* = − 6.15, *p* < 0.001; PwVD: Z = − 5.14, *p* < 0.001) in both groups. Substantial within-group differences for DTC were noted for both groups between FGA-M with FGA-N (Healthy: *Z* = − 6.36, *p* < 0.001; PwVD: Z = − 4.62, *p* < 0.001) and FGA-L (Healthy: *Z* = − 5.93, *p* < 0.001; PwVD:* Z* = − 4.45, *p* < 0.001). After Bonferroni correction is applied, the within-group differences observed only for PwVD between FGA with FGA-M (*Z* = − 2.67, *p* = 0.008;) and between FGA-L and FGA-N (Z = − 2.47, *p* = 0.013) were no longer significant.

### CANTAB and self-report measures

Between-group differences (Table 3) were noted for spatial working memory (U = 783.00, z = − 2.975, *p* = 0.003, *r* = 0.28), paired associates learning (U = 897.00, z = − 2.18, *p* = 0.029, *r* = 0.22) and reaction time (*U* = 904.50, *z* = − 2.12, *p* = 0.034, *r* = 0.21). After applying the Bonferroni correction, the between-group difference remains significant only for spatial working memory. No significant between-group differences were noted for delayed matching to (U = 1002.00, z = − 1.45, *p* = 0.147, *r* = 0.14) and rapid visual information processing (U = 1025.50, z = − 1.16, *p* = 0.246, *r* = 0.12). Between-group differences (*p* < 0.001) were also observed for all self-report measures (Table 3): balance confidence (ABC, U = 241.50, z = − 6.76, *r* = 0.67), dizziness handicap total score (DHI total, U = 2.00, z = − 9.06, *r* = 0.90), vestibular symptoms (VSSV, U = 33.50, z = − 8.43, *r* = 0.84), autonomic symptoms (VSSA, U = 243.50, z = − 6.65, *r* = 0.66), visual induced dizziness (SCQ, U = 125.00, z = − 7.59, *r* = 0.76). anxiety (HADS-A, U = 528.50, z = − 4.76, *r* = 0.47) and depression (HADS-D, U = 346.00, z = − 6.08, *r* = 0.60).

### Correlations

Only correlations that remain significant after application of the Bonferroni correction are reported. In healthy participants’, increasing age was associated with poorer performance on mean scores for delayed matching to sample (*r* = − 0.43, *p* = 0.001) and rapid visual information processing (*r* = 0.37, *p* = 0.003). In PwVD, after applying the Bonferroni corrected alpha level, no significant correlations were identified between cognitive function scores and other variables.

### Multiple linear regression analysis for FGA single and DT predictive models

#### Healthy participants

For all FGA models, assumptions of linearity, homoscedasticity, multicollinearity, normality, and independence of residuals (Durbin-Watson: FGA = 1.78, FGA-M = 2.03, FGA-L = 2.05, FGA-N = 2.22) were confirmed. Outliers with studentized deleted residuals >  ± 3 SD were identified for FGA single (n = 3), FGA-M (n = 2), FGA-N (n = 2) and FGA-L (n = 4) and were excluded from multiple regression analyses.

Spatial working memory and simple reaction time significantly predicted FGA single, F(2,55) = 9.20, adjusted R^2^ = 0.22,* p* < 0.005 and FGA-N, F(2,56) = 10.06, adjusted R^2^ = 0.24, *p* < 0.005 (Table 4). Age was the only significant predictor for FGA-M, F(1,57) = 10.11, adjusted R^2^ = 0.14, *p* = 0.002 (Table 4). Sex and paired associates learning explained 18% of the variance for FGA-L, F(2,55) = 7.39, adjusted R^2^ = 0.18, *p* = 0.001 (Table 4). Variables added significantly to the prediction model for FGA single and all FGA DT conditions (*p* < 0.05).

**Table 4:** Multiple regression results for FGA single and dual-task conditions for healthy participants.

	*B*	95% CI for *B*		*SE B*	β	*R*^2^	Δ*R*^2^
		*LL*	*UL*				
FGA							
Model						0.25	0.22
Constant	33.14***	30.99	35.29	1.07			
RTI	− 0.01***	− 0.02	− 0.01	0.00	− 0.43***		
SWM	− 0.03***	− 0.04	− 0.01	0.01	− 0.45***		
FGA-M							
Model						0.15	0.14
Constant	30.22***	29.30	31.14	0.46			
Age	− 0.03**	− 0.04	− 0.01	0.01	− 0.39**		
FGA-N							
Model						0.26	0.24
Constant	36.29***	30.03	42.54	3.12			
RTI	− 0.03**	− 0.05	− 0.01	0.01	− 0.35**		
SWM	− 0.09***	− 0.13	− 0.05	0.02	− 0.51***		
FGA-L							
Model						0.21	0.18
Constant	24.84***	22.37	27.03	1.23			
Gender	1.66*	0.19	3.13	0.73	0.27*		
PAL	− 0.78*	− 0.13	− 0.03	0.02	− 0.39*		

Model, “Backward” method in SPSS statistics 27; *B*, unstandardised regression coefficient; CI, confidence interval; *LL*, lower limit and *UL*, upper limit; *SE B*, standard error of the coefficient; β, standardized coefficient; *R*^2^, coefficient of determination; Δ*R*^2^, adjusted *R*^2^; FGA, Functional Gait Assessment; FGA- M, -N and -L, FGA performed simultaneously with motor, numeracy or literacy task; RVP, Rapid Visual Information Processing; PAL, Paired Associates Learning; RTI, reaction time; ABC, Activities of Balance Confidence Scale. *p ≤ 0.05 **p ≤ 0.01 ***p ≤ 0.001.

### PwVD

For all FGA models, assumptions of linearity, homoscedasticity, normality and independence of residuals (Durbin-Watson: FGA = 2.07, FGA-M = 2.47, FGA-L = 2.08, FGA-N = 1.5) were confirmed. Multicollinearity was violated with correlations > 0.7 between ABC with DHI total score, DHI total score with HADS-D, HADS-A with HADS-D, SCQ with VSSA, VSSV with VSSA resulting in DHI total score, HADS-D and VSSA being excluded from all models. Outliers with studentized deleted residuals >  ± 3 SD were identified for FGA single (n = 2), FGA-M (n = 2) and FGA-N (n = 1) and FGA-L (n = 1) and were excluded from the relevant multiple regression analyses.

Rapid visual information processing, paired associates learning, ABC, age, sex and hearing loss significantly predicted FGA single, F(5, 31) = 23.92, *p* < 0.001, adjusted R^2^ = 0.76 (Table 5) and FGA-M, F(5,32) = 22.74, adjusted R^2^ = 0.76,* p* < 0.001 (Table 5). Rapid visual information processing, paired associates learning, ABC, sex and migraine significantly predicated FGA-L in the initial model, F(5,32) = 16.04, adjusted R^2^ = 0.67, *p* < 0.001 while rapid visual information processing, paired associates learning, delayed matching to sample, sex, and migraine significantly predicted FGA-N, F(5,32) = 15.61, adjusted R^2^ = 0.66, *p* < 0.001 (Table 5). All variables added significantly to the prediction models for each FGA single and DT condition (*p* < 0.05).

**Table 5:** Multiple regression results for FGA single and dual-task conditions for PwVD.

	*B*	95% CI for *B*		*SE B*	β	*R*^2^	Δ*R*^2^
		*LL*	*UL*				
FGA							
Model						0.79	0.76
Constant	40.57***	34.37	46.76	3.04			
Gender	− 2.18**	− 3.84	− 0.52	0.82	− 0.24**		
Age	− 0.08**	− 0.14	− 0.03	0.03	− 0.27**		
RVP	− 0.03***	− 0.03	− 0.02	0.00	− 0.67***		
PAL	− 0.05*	− 0.09	− 0.00	0.02	− 0.19*		
ABC	0.04**	0.01	0.07	0.01	0.25***		
FGA-M							
Model						0.78	0.76
Constant	43.06***	35.33	50.78	3.79			
Gender	− 3.26**	− 5.32	− 1.21	1.01	− 0.29**		
Age	− 0.12***	− 0.18	− 0.05	0.03	− 0.31***		
RVP	− 0.02***	− 0.03	− 0.02	0.00	− 0.25***		
PAL	− 0.09**	− 0.14	− 0.03	0.03	− 0.30**		
ABC	0.05**	0.01	0.08	0.02	0.53**		
FGA-N							
Model						0.71	0.66
Constant	28.19***	14.64	41.75	6.86			
Gender	− 4.37**	− 6.98	− 1.75	1.28	− 0.35**		
RVP	− 0.03***	− 0.04	− 0.02	0.01	− 0.52***		
PAL	− 0.09**	− 0.15	− 0.02	0.03	− 0.27**		
DMS	0.12*	0.01	0.23	0.05	0.23*		
Migraine	3.09 **	0.71	5.47	1.17	0.26**		
FGA-L							
Model						0.72	0.67
Constant	35.27***	26.27	44.27	4.42			
Gender	− 4.51**	− 7.19	− 1.84	1.31	− 0.36**		
RVP	− 0.03***	− 0.04	− 0.02	0.01	− 0.51***		
PAL	− 0.08*	− 0.15	− 0.01	0.03	− 0.25*		
ABC	0.05*	0.01	0.10	0.02	0.25*		
Migraine	3.17**	0.83	5.50	1.15	0.27**		

Model, “Backward” method in SPSS statistics 27; *B*, unstandardised regression coefficient; CI, confidence interval; *LL*, lower limit and *UL*, upper limit; *SE B*, standard error of the coefficient; β, standardized coefficient; *R*^2^, coefficient of determination; Δ*R*^2^, adjusted *R*^2^; FGA, Functional Gait Assessment; FGA- M, -N and -L, FGA performed simultaneously with motor, numeracy or literacy dual-tasks; DMS- Delayed Matching to Sample; RVP, Rapid Visual Information Processing; PAL, Paired Associates Learning; RTI, reaction time; ABC, Activities of Balance Confidence Scale. *p ≤ 0.05 **p ≤ 0.01 ***p ≤ 0.001.

Regression coefficients and standard errors are in Tables 4 and 5 for healthy participants and PwVD, respectively.

## Discussion

This study investigated the effect of motor and cognitive DTs on complex gait performance, as assessed by FGA, in both PwVD and healthy participants. All PwVD had chronic symptoms and our findings are only applicable to this population. Results showed more pronounced FGA DTCs for cognitive (numeracy and language) versus motor tasks in both groups. Rapid visual processing, paired associates learning, and gender were predictors of FGA performance under both single and DT conditions in PwVD.

### Cognitive function in PwVD and its association with functional gait

Impaired spatial working memory was noted for PwVD compared to healthy participants, which is in agreement with findings reported in previous work in persons with unilateral vestibular loss^41^. Our finding of impaired paired associates learning in PwVD compared to healthy participants was no longer significant following Bonferroni correction. This may be due to under powering of the study. Worse performance for paired associated learning in PwVD is reported in the animal literature; rats use self-motion (vestibular) signals to disambiguate between spatial locations to form object-place associations^42^. The pathway involved may include the medial temporal lobe, which is responsible for the visually induced self-perception of motion^43^ and is activated by sensory conflict between visual and vestibular stimuli^44^. Interestingly, bilateral vestibular deafferentation leads to changes in the biogenic amine pathways (serotonin/tryptophan) of the medial temporal lobe^45^. It is an intriguing question whether worse paired associates learning scores in PwVD versus healthy participants may be part of vestibular sensory driven cognitive decline, but both the presence of worse learning scores in PwVD and such a potential association would need to be further investigated. Paired associates learning also predicted all FGA single and DT conditions in PwVD and FGA-L in healthy participants possibly because both gait and an object-space association memory task are dependent on self-motion vestibular (idiothetic) perception^42^.

Given the predictive role of specific cognitive domains for FGA single and DT scores, an area of potentially fruitful research would be studying whether vestibular rehabilitation has positive cognitive effects. In older adults, physical combined with cognitive training resulted in a greater improvement for paired associates learning versus cognitive training alone^46^. Vestibular rehabilitation incorporating progressively more challenging functional gait exercises could lead to improved paired associates learning by promoting enhanced perception of self-motion during locomotion and even by possible direct medial temporal lobe activation^47^. Recently, two exploratory studies assessed vestibular rehabilitation outcomes, in isolation or with the use of a virtual reality head mounted display, in older adults with and without MCI (mild cognitive impairment) and unilateral vestibular hypofunction^48,49^. The findings suggest that people with MCI benefit from vestibular rehabilitation, with improvements noted in functional gait, postural sway, self-perceived handicap from dizziness and/or quality of life. It appears that vestibular hypofunction is more prevalent in older adults with Alzheimer’s Disease^50^. Klatt et al.^51^ state that vestibular rehabilitation might be able to improve balance, and decrease falls, health care costs, and caregiver burden for people with cognitive impairment and have proposed a theoretical and practical guide for vestibular rehabilitation in this population.

Rapid information processing was identified as a predictor for all FGA single and DT models in PwVD while reaction time was not. Reaction time is a simple visual task response latency, while rapid visual information processing is the response speed for detecting a target number sequence. The reaction time task is reliant on dopamine pathways as the dopamine 4 receptor gene and a DRD4 polymorphism is associated with attentional disorders^52^, while rapid visual information processing is reliant on the cholinergic pathway^53^.

Current findings for PwVD indicate that dynamic gait +/− dual tasking is more “effortful” for this population^1^. Paired associates learning and rapid visual information processing which are related to perception and sensory stimuli predicted FGA single and all DT conditions. These changes in cognitive domains may delay PwVD’s ability to encode and embed new information in memory during tasks that require a less practiced task strategy, thus having a negative impact on FGA and DT performance.

In healthy participants, spatial working memory and reaction time predicted FGA single and FGA-N. A small association has previously been observed between gait and spatial working memory^54^ while processing speed has been shown to contribute to stepping errors^55^ and stride length^56^ in older adults. In our study, the predictive role of these cognitive domains is present irrespective of age. However, predictive model effect sizes were weak for healthy participants and results must be considered with caution.

### The relationship between FGA scores, age, and sex

Increasing age is associated with poorer FGA performance in healthy adults^28^. The weak predictive role for age in healthy participants is likely due to the predominantly younger age of these individuals in the current versus previous studies^28,57^. Age predicated FGA single and the FGA motor DT condition with older adults having worse scores in PwVD; however, age was not identified as a predictor for cognitive FGA DT conditions. Similar findings have previously been reported whereby the gait DTC increases more in younger versus older adults for a numeracy DT activity^58^. The authors hypothesised that older adults may have reached their maximal resource capacity with the numeracy task and subsequently showed minimal changes in the gait speed DTC as task difficulty increased from counting backwards in 3’s to 7’s^58^. It has been shown that compared to younger adults, older persons consume more neural resources to perform simple tasks^58^ and are therefore likely to achieve the ceiling effect under high demand conditions^58,59^. We hypothesise that in PwVD the ceiling effect for age was achieved with the FGA in isolation and therefore the further impact of age on numeracy and literacy DT FGA conditions was insignificant.

Sex was a predictive factor for all FGA conditions in PwVD with females having worse FGA single and DT scores. Sex specific gait strategies in response to a physiologic impairment have also been observed. Age-related decreases in saccular function are associated with an increase and decrease in gait speed for men and women, respectively, which may explain the sex impact in all FGA conditions for PwVD^60^. Recently though significant magnitude and pattern differences in hip, knee and ankle kinematics and kinetics have been reported for healthy women and men, of varying ages, between 20 and 75 years old^61^. The differences in gait kinetics and kinematics were irrespective of age category and the findings suggest that it is important to consider sex-specific analyses in gait studies^62^. It may be that kinematic and kinetic sex-specific differences have a distinct impact on functional gait in PwVD and further work is required to assess the impact of sex on functional single and DT gait in this population.

In healthy participants, sex was a predictive factor for FGA-L with women achieving better FGA scores. Sex specific differences have been reported in healthy adults for verbal fluency tasks that require participants to switch categories, with women performing better than men^60^. The literacy task involved switching between reciting alternate alphabet letters, months or days of the week. Thus, the literacy task may have been easier for healthy women versus men resulting in less DT interference and better FGA scores for the former.

### Balance confidence and FGA performance

Balance confidence independently predicted FGA performance, except FGA-N in PwVD. Decreased balance confidence in performing functional activities is associated with actual balance performance in older adults with vestibular dysfunction^63^. Balance confidence contributes to self-efficacy, a person’s belief in their ability to succeed in a particular situation^64^. Self-efficacy plays an important role in the effort applied to a task and stress experienced when presented with a challenge^64^. Persons with decreased balance confidence and self-efficacy may modify their behavior to avoid activities and situations that increase symptoms and/or falls risk^64^. PwVD avoid head movement, physical activity, travel, and social commitments to mitigate symptoms^63^. Thus, understanding the relationship between balance confidence with FGA and specific cognitive domains, and addressing this together with self-efficacy should be an important interventional target which may result in improved management.

Self-efficacy has been identified as a mediator of the relationship between cognitive ability and conscientiousness with performance^65^. However, the magnitude of these relationships varies with task complexity. Self-efficacy has been found to mediate the relationships of cognitive ability and conscientiousness with performance on less challenging tasks, but not on more complex tasks^65^. Chen et al.^65^ suggest that as tasks become broader and more complex, more generalized, individual, differences influence task performance better than task-specific constructs, such as self-efficacy. This may explain why balance confidence did not predict FGA-N, the most challenging DT condition, in the current study.

### Migraine and FGA performance

Migraine independently predicted FGA-N and FGA-L, with the positive β coefficient for migraine (Table 5) suggesting that it is associated with higher (i.e., better) FGA scores. Although cognitive deficits particularly for memory and attention have frequently been reported during the pre-ictal and ictal migraine phase^66,67^, findings during the interictal migraine phase have been divisive^66,68,69^. Factors including migraine frequency, psychological comorbidity, and sample size may account for the heterogeneity of these findings with those experiencing more frequent migraines together with increased psychological symptoms performing worse^67^. A recent study described better performance for visuospatial memory and learning abilities in persons with migraine compared to healthy controls indicating a “cognitive advantage” in those with migraine who have a low frequency of attacks per month^66^, as in our study. Better scores for cognitive task performance have been reported in middle age and older adults with migraine for both global measures of cognition and domain specific findings for executive function and susceptibility to interference^70^. In the present study, none of the persons with migraine history experienced > 3 migraines per month and anxiety and depression symptom scores were within normal levels, which may have contributed to migraine being identified as an independent predictor for FGA-N and FGA-L. However, the sample size for persons with migraine is small and further work is needed to confirm these findings.

### The impact of motor, numeracy, and literacy DTs on FGA performance

Average FGA single and DT scores were significantly worse in PwVD compared to healthy participants in our study and to age-range normative values for the FGA single published previously^28^. Cut-off scores for falls risk for the FGA single or DT conditions have not been determined in this population. However, a 20% or greater DTC for gait velocity has a destabilizing effect and increases falls risk^71^. The DTC for FGA-N surpassed this percentage threshold and approached it for FGA-L, suggesting an increased falls risk for these DT conditions compared to FGA single and FGA-M. This finding has potential implications for clinical practice where DT functional gait is often not considered within assessment and intervention programs. Currently, no studies in PwVD have included a pre-post intervention DT gait assessment or investigated the impact of DT training on vestibular rehabilitation outcomes.

DT gait assessment sensitivity depends on the cognitive task used. For both groups, the numeracy and literacy tasks incurred significant DTCs in functional gait compared to the motor task, with the highest sensitivity noted for the numeracy task, as has been reported in persons with MCI^72^ and healthy older adults^73^. Literacy (i.e., superior part of Broca's area and premotor cortex) and numeracy task (i.e., temporo-parietal regions) cortical networks are distinct. The numeracy, relative to a literacy task may share more cortical networks with gait, thus producing greater changes in FGA performance^73^. It has been suggested that the left posterior parietal cortex may be involved in sensorimotor integration processes and gait control in real-world conditions^74^, while in older adult females, temporal lobe activation, especially the hippocampus, is associated with gait adaptability during unaccustomed treadmill walking^75^. However, in the current study, although the numeracy task had the highest DTC, no significant differences were noted between FGA-N and FGA-L in the healthy group and the difference between the two was no longer significant in PwVD after Bonferroni correction was applied. The high variability noted for the numeracy and literacy DTCs for both groups, as well as the predominantly younger age for the healthy group, may have contributed to this finding.

Tasks differ in their ability to challenge gait^14,58,72^. Although, in our study, FGA-N had the highest DTC, a low demand numeracy task (i.e. subtracting from 1) may have shown similar results to FGA single as it is more rhythmic and can cue step pattern^72^. The motor task had the least impact on FGA performance and only a minimal effect on DTC in both groups. Walking while carrying a cup of water has been conceptualized as a single, complex task with one action goal which is to transport the water without any spills^14^. Postural control requirements for gait cannot be dissociated from those for holding a cup of water, as control for transporting a hand-held object while walking is contingent upon the inertial forces created by the gait cycle which act on the object^14^. The cup of water represents an additional postural constraint which increases task complexity but not the number of tasks performed and is therefore insufficient to reveal a DT interference effect^14^. A suitable motor task would be walking while texting on a mobile phone whereby each task goal is separable and can be distinctly measured^14^.

Performance decrements during DT gait are also associated with a person’s ability to allocate cognitive resources which depends on cognitive task type and gait task complexity^14^. Task complexity is determined not only by the task’s difficulty level but also the performer’s expertise and abilities^14^. Thus, a further factor which may have contributed to outcome for all participants was each person’s experience with performance of the particular tasks, which was not quantified.

The current study provides insights into the effect of varying task types on FGA performance in PwVD. Further work is needed to determine the optimum task type and content for gait assessment in PwVD. Predictive factors show similarities and differences for DT FGA conditions indicating various DT conditions should be assessed to identify the most appropriate tasks. The proposed DT difficulty framework^76^ for persons with MCI can be implemented for PwVD and used to guide clinicians in choosing appropriate tasks to progressively increase cognitive challenge to identify deficits^76^. The poorer performance for specific cognitive domains and their impact on single and DT FGA performance, particularly for FGA-N and FGA-L, indicates a need for cognition and functional gait in combination with a cognitive task to be included within a clinician’s assessment in PwVD. In persons who experience dizziness and balance problems following a mild traumatic brain injury, subjective cognitive function scores significantly improve pre-post vestibular rehabilitation, although cognitive symptoms persist^77^. No studies in PwVD have included cognitive function tests pre-post treatment nor is cognition specifically targeted within published vestibular rehabilitation studies. A cognitive and DT FGA assessment may allow for provision of targeted interventions and improved outcomes in future.

### Study limitations

Some study limitations are present. Baseline cognitive data was not collected; therefore, DTC on the cognitive task cannot be determined. As secondary task category and content impacts on outcome, future studies in PwVD should investigate the effect of tasks of varying difficulty within categories including auditory tasks. Passive listening to multi-talker babble noise affects FGA performance in young and particularly older adults and those with decreased hearing capacity^19^. Poor sleep quality^78^ and low physical activity levels^79^ have a detrimental impact on gait; these factors were not included, however, their impact on single and DT FGA should be considered clinically and in future work.

## Conclusion

This study provides insights into the effect of chronic vestibular disorders on cognition and DT functional gait. Clinicians should be aware of the additional negative impact of literacy and numeracy tasks on functional gait performance in PwVD. In PwVD, poorer cognitive scores are noted for paired associates learning, reaction time and spatial working memory, irrespective of age, albeit after Bonferroni correction, only the latter remained significant. Gender, varying cognitive domains, balance confidence and migraine history predict FGA single and/or DT performance. The findings support inclusion of a multiple domain cognitive measure and DT FGA that considers various tasks to identify each person’s deficits for the provision of targeted interventions towards optimal management and outcome in PwVD.

## Data Availability

Anonymized data will be available by request from qualified researchers whose data use has been approved by an independent review committee. Initial requests should be addressed to Dr Marousa Pavlou at marousa.pavlou@kcl.ac.uk.
